# The Role of TGF-β in Bone Metastases

**DOI:** 10.3390/biom11111643

**Published:** 2021-11-06

**Authors:** Trupti Trivedi, Gabriel M. Pagnotti, Theresa A. Guise, Khalid S. Mohammad

**Affiliations:** 1 Department of Endocrine Neoplasia and Hormonal Disorders, Division of Internal Medicine, The University of Texas MD Anderson Cancer Center, Houston, TX 77030, USA; TTrivedi@mdanderson.org (T.T.); GMPagnotti@mdanderson.org (G.M.P.); TAGuise@mdanderson.org (T.A.G.); 2 College of Medicine, Alfaisal University, Riyadh 11533, Saudi Arabia

**Keywords:** bone metastases, transforming growth factor-β (TGF-β), programmed cell death ligand (PD-L1), immune cells, TGF-β therapeutic targets, check-point inhibitors, bone resorption

## Abstract

Complications associated with advanced cancer are a major clinical challenge and, if associated with bone metastases, worsen the prognosis and compromise the survival of the patients. Breast and prostate cancer cells exhibit a high propensity to metastasize to bone. The bone microenvironment is unique, providing fertile soil for cancer cell propagation, while mineralized bone matrices store potent growth factors and cytokines. Biologically active transforming growth factor β (TGF-β), one of the most abundant growth factors, is released following tumor-induced osteoclastic bone resorption. TGF-β promotes tumor cell secretion of factors that accelerate bone loss and fuel tumor cells to colonize. Thus, TGF-β is critical for driving the feed-forward vicious cycle of tumor growth in bone. Further, TGF-β promotes epithelial-mesenchymal transition (EMT), increasing cell invasiveness, angiogenesis, and metastatic progression. Emerging evidence shows TGF-β suppresses immune responses, enabling opportunistic cancer cells to escape immune checkpoints and promote bone metastases. Blocking TGF-β signaling pathways could disrupt the vicious cycle, revert EMT, and enhance immune response. However, TGF-β’s dual role as both tumor suppressor and enhancer presents a significant challenge in developing therapeutics that target TGF-β signaling. This review presents TGF-β’s role in cancer progression and bone metastases, while highlighting current perspectives on the therapeutic potential of targeting TGF-β pathways.

## 1. Introduction

Cancer is a major cause of death in the United States and worldwide [1,2]. Several types of cancers have high propensity to metastasize to bone. These bone metastases are associated with skeletal complications, including pain, pathological fracture, hypercalcemia, and spinal cord compression [3,4]. Collectively, these comorbidities are defined as skeletal-related events. Once cancer spreads to bone, it is incurable and contributes significantly to cancer-associated morbidity and, also, increase healthcare cost [3,5,6,7,8].

Anti-resorptive drugs are a standard treatment modality that reduces skeletal morbidity and delays skeletal-related events but does not cure the disease [9,10,11]. Due to significant advancements in diagnosis and treatment, patients with bone metastases, particularly from breast cancer and prostate cancer, can live longer after diagnosis. However, they suffer from debilitating complications of skeletal-related events, which significantly increase morbidity [12]. Better treatment options with a long-term goal of disease prevention and cure are needed to reduce the morbidity associated with bone metastases.

**Figure 1:**
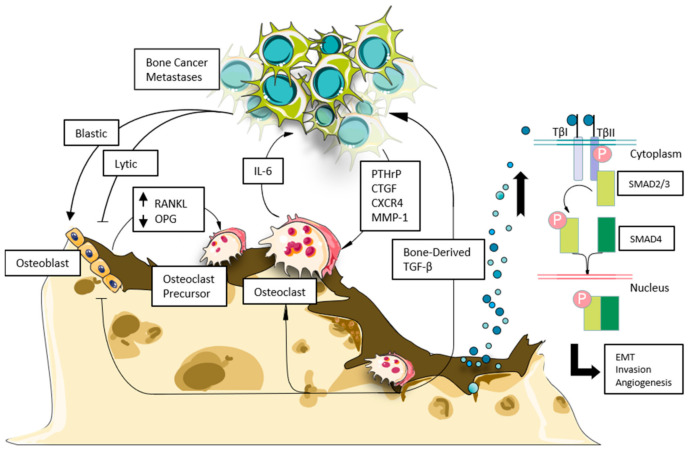
Interaction of TGF-β and the tumor–bone marrow microenvironment. Osteoclast activity is elevated by interaction with inflammatory factors secreted from metastatic cancer cells in the marrow. Heightened osteoclast-mediated bone resorption releases the TGF-β contained within the bone matrix, inducing cancer cell proliferation. This effect can have profound impact on osteoblasts, depending on the nature of invading tumor cells (breast cancer metastases release predominantly osteoblast inhibitory factors), while prostate cancer metastases tend to promote uncontrolled osteoblast-driven bone formation). Invading tumor cells fuel osteoclast activity as well, causing imbalances in bone remodeling to favor osteoclast-mediated bone resorption (increased RANKL), local inflammation, and increased tumor burden. Binding of excess TGF-β to its type I and II receptors phosphorylates the cytoplasmic domain, releasing SMAD2/3 from the TGF-β receptor complex to bind to SMAD4. The SMAD 2/3/4 complex then translocate to the nucleus to promote epithelial-mesenchymal transition, tumor cell invasion, and angiogenesis.

The bone microenvironment is a unique milieu that provides fertile soil for cancers to thrive (Figure 1) [7]. Several hormones, cytokines, and growth factors are stored in the mineralized bone matrix and released in response to osteoclastic bone resorption [12,13] (Figure 1). Transforming growth factor β (TGF-β) is one of the major bone-derived factors and is a member of the TGF-β superfamily, which also includes activins, inhibin, and bone morphogenetic proteins (BMPs) involved in the bone remodeling process [14,15]. TGF-β releases as an inactive precursor form in which it remains bound with a latency-associated peptide that keeps it in an activated state. During bone resorption, active TGF-β is released due to pH changes in the local environment and proteolytic cleavage from latent peptides [9]. In addition to its role in bone remodeling, TGF-β also triggers the release of pre-osteolytic and osteolytic factors from tumors that further stimulate bone resorption (Figure 1) [16,17].

Owing to its dual effects on both bone and cancer cells, TGF-β is considered an important driver of the feed-forward vicious cycle of tumor progression in bone (Figure 1) [18]. Thus, blocking TGF-β release, secretion, and/or signaling could be a promising therapeutic strategy for the treatment of devastating bone metastases. For the past two decades, multiple strategies have been used to target TGF-β signaling pathway such as TGF-β receptor kinase inhibitors, TGF-β neutralizing antibodies, soluble receptor decoys, and TGF-β antisense oligonucleotides, which could inhibit TGF-β activity [19,20]. These therapeutic strategies have successfully shown their benefit in in vitro studies and preclinical animal models. However, in clinical trials the survival benefits with TGF-β signaling targets are limited and sometimes associated with adverse effects [21,22]. In such situation combination treatment strategies with bone targeted therapeutics may have potential benefits.

In recent years cancer immunotherapy has brought revolutionary change in the cancer treatment. A cluster of patients with advanced cancer and distant metastasis who did not show response to traditional chemotherapy, radiation therapy, or signaling pathway inhibitors have shown significant improvement in disease prognosis after immunotherapy use. Despite the huge progress in the immunotherapy, several clinical trials have reported that the use of immune checkpoint inhibitors is limited to specific cancer types such as melanoma and non-small cell lung carcinoma [23,24]. A preclinical study has shown resistance to immune checkpoint inhibitors in prostate cancer bone metastases via suppression of CD4 Th1 effector T cell response and clonal expansion of CD8T cells [24,25]. Due to increased bone resorption and increased TGF-β release restrained Th1 lineage development, and thus blocking TGF-β signaling together with immune checkpoint inhibitors could have better effects on regression of prostate cancer bone metastases [26].

From the previous studies, current studies and results from clinical trials it seems that synergistic therapeutic approach by combining TGF-β inhibitors with immunotherapy can have better therapeutic outcome in patients with bone metastases as highlighted in this review.

## 2. Physiology of Bone Remodeling

Collagen matrix is a major component of bone and is mineralized by hydroxyapatite. Bone tissue comprises 60% minerals, 30% organic matrix, and 10% water. The mineral part is composed of hydroxyapatite crystals made up of calcium and phosphate. The inorganic matrix is made up of 98% type I collagen and 2% non-collagenous protein, including growth factors, cytokines, and extracellular matrix proteins such as osteonectin, osteopontin, bone sialoprotein, osteocalcin, and proteoglycans. Even though non-collagenous protein occupies only 2% of bone tissue, it contributes to the biological function and regulation of bone and some non-bone tissues [27]. Growth factors, hormones, and cytokines including TGF-β, BMPs, OPG, osteocalcin, IGF, interferon-γ, TNFs, and ILs are embedded in the mineralized bone matrix and actively involved in the regulation of bone cell differentiation and activation to maintain overall bone homeostasis [27,28].

### 2.1. Bone Cells

Three major cell types regulate bone function: bone-resorbing osteoclasts, bone-forming osteoblasts, and the cells embedded in the bone matrix, the osteocytes.

#### 2.1.1. Osteoclasts

Osteoclasts arise from the hematopoietic progenitors that also make monocyte/macrophage lineages. Two osteoblast-derived cytokines, namely RANKL and macrophage colony-stimulating factor, are key signals that induce recruitment and differentiation of pre-osteoclasts into osteoclasts. The same signals are responsible for the activation and survival of mature, resorbing osteoclasts [29,30]. Specifically, RANKL induces osteoclast differentiation and activation through binding to its cognate receptor, RANK, on the surface of hematopoietic precursor cells and osteoclasts [31,32]. However, some studies indicate that osteocytes also express large amounts of RANKL, which at certain stages may be greater than what is released by osteoblasts [33,34], and which imparts osteocytes with the capacity to support osteoclastogenesis [33,35]. Both RANKL and macrophage colony-stimulating factor are necessary for the induction of genes that classify the osteoclast lineage, including those encoding tartrate-resistant acid phosphatase (TRAP), cathepsin K (CATK), calcitonin receptor, and αβ_3_ integrin [29]. Osteoclast function is regulated by the intricate interaction of OPG (a soluble decoy receptor for RANKL) and RANK and their common ligand, RANKL [30,36]; OPG inhibits RANKL binding to RANK and thereby blocks osteoclastic bone resorption. The ratio of RANKL to OPG thus determines the level of osteoclastogenesis [36,37,38]. Therefore, expression levels of RANKL and OPG are modulated concurrently to regulate bone resorption by controlling the activation of RANK on osteoclasts [29,30,39,40]. Systemic bone-resorbing factors such as parathyroid hormone, interleukin-1 IL-1, and TNF-α can increase the expression of RANKL relative to that of OPG by osteoblasts, thereby indirectly promoting osteoclast activity [12,13].

#### 2.1.2. Osteoblasts

Osteoblasts are the cells derived from mesenchymal stem cells. After progressing through several stages of differentiation, these osteoprogenitor cells mature to become fully functional osteoblasts, which then synthesize and secrete type I collagen, non-collagenous matrix proteins (such as osteocalcin, osteopontin, or bone sialoprotein), and enzymes required for mineral deposition (e.g., alkaline phosphatase) [41,42,43].

#### 2.1.3. Osteocytes

Osteocytes are terminally differentiated osteoblasts integrated in the bone matrix. Structurally, osteocytes are smaller than osteoblasts and occupy lacunae in mineralized bone. Osteocytes have a role in phosphate homeostasis and assist in bone resorption by flagging osteoclasts to sites of microdamage and osteocyte apoptosis [42,43,44,45].

### 2.2. Bone Remodeling

Bone is a dynamic tissue that undergoes constant remodeling after modeling is complete. Longitudinal and radial growth of bone occurs during childhood and adolescence [41,46]. The process of bone remodeling is achieved through the coupled actions of osteoclasts and osteoblasts [42]. The function of osteoclasts in this process is to remove old bone so that osteoblasts can replace it with new bone. The initial step of bone resorption is removing unmineralized osteoid (newly formed matrix) through proteolytic digestion of the collagenous matrix by bone-lining cells. This step exposes the underlying mineralized matrix for osteoclast access [47]. Upon initiation of osteoclastogenesis, mononuclear precursor cells are recruited and fused into multinucleated pre-osteoclasts, which bind to the bone matrix via integrin receptors. At the same time, RANKL expressed by osteoblasts binds to RANK on precursor and mature osteoclasts. Additionally essential for the induction of osteoclastogenesis are locally released bone matrix–derived components such as IGF-1 and soluble factors such as IL-1 [44]. Following the adhesion of osteoclasts to the bone surface, the cells form a sealing zone and develop highly convoluted finger-like projections, known as the “ruffled border” [48,49]. A hydrogen ion transporting machinery at this specialized cell membrane creates an acidic environment by lowering the pH in the resorption lacunae, which leads to the dissolution of the inorganic matrix [50]. Subsequently, vesicle-bound CATK, reactive oxygen species generated by TRAP, MMP-9, MMP-13, and other collagenases are secreted onto the bone surface, resulting in the breakdown of the organic bone matrix [41,48,51]. As a result of these coordinated processes, a bone resorption pit develops, and minerals and proteolytic fragments are released from the extracellular matrix. The mobilized minerals and proteins are absorbed by endocytosis and, following further metabolism, are further released into the extracellular fluid [41,43]. A negative-feedback mechanism involving factors such as TGF-β and high extracellular calcium concentrations regulates osteoclast activity, which ultimately leads to osteoclast apoptosis [47,52]. Following osteoclast death and removal, osteoblast precursors are recruited in preparation for osteoblastic bone formation [47]. Thus, at the completion of bone resorption, the resorption cavities contain monocytes, osteocytes (released from the bone matrix), and pre-osteoblasts. Under normal circumstances, bone resorption and bone formation are closely coupled through the action of specific cellular and matrix-derived signals such as TGF-β, IGF-1 and -2, BMPs, platelet-derived growth factor, and fibroblast growth factor [43,44]. During bone formation, osteoblasts synthesize a collagen matrix and regulate its mineralization by releasing small membrane-bound vesicles, which concentrate calcium and phosphate and degrade inhibitors of mineralization [41,42]. After that, 50–70% of osteoblasts undergo apoptosis while the remaining cells become osteocytes and bone-lining cells. Once the newly formed matrix (osteoid) has been fully mineralized and converted to the bone lamella, the bone remodeling is complete [42,44,53,54].

## 3. TGF-β Signaling Mechanisms

TGF-β is a multifunctional cytokine that was historically named for its ability to transform normal fibroblast behavior. Almost all tissues express TGF-β both at developmental stages and throughout adult life [55]. In many tissues, TGF-β plays a dual role; for example, it promotes fibroblast growth and inhibits their proliferation [56]. TGF-β orchestrates the response to tissue injury by mediating tissue repair via inducing epithelial-to-mesenchymal transition (EMT); it also promotes cell migration and regulates immune responses. Several diseases, including chronic fibrosis, inflammation, and cancer, are associated with the dysregulation of the normal function of TGF-β [57].

### 3.1. The TGF-β Superfamily

The TGF-β superfamily comprises 32 ligands that are grouped into the TGF-β and BMP subfamilies based on sequence similarity and function [58]. The TGF-β subfamily comprises three TGF-β ligands (TGF-β1, TGF-β2, and TGF-β3), two activins (A and B), NODAL, GDF1, GDF3, GDF8 (also known as myostatin), GDF9, and GDF11. The BMP subfamily includes 10 BMPs, several GDFs, and the anti-Müllerian hormone. Additional members of this superfamily encode antagonistic ligands and a few distant outliers. All three TGF-βs and especially TGF-β1 are essential for immune regulation [59]. In the mammalian genome, the three TGF-βs acts as disulfide-linked dimers [60,61]. Each TGF-β gene encodes a precursor protein with a short N-terminal signal peptide required for secretion and a C-terminal mature TGF-β polypeptide. These two domains are linked by a long pro-segment cleaved during secretion but remain associated with mature TGF-β to act as a chaperone. TGF-β is secreted as a latent form, and after secretion, the pro-domain (latency-associated peptide, LAP) keeps TGF-β in an inactive state. Consequently, the mature TGF-β dimer is secreted as a latent complex with two copies of a non-covalently associated pro-segment, which prevents TGF-β from binding to its cell-surface receptors [17,62]. TGF-β transmits signals through two transmembrane serine-threonine kinase receptors, type I and type II (TβRI and TβRII) [61]. Seven type I receptors ALKs 1-7, and five type II receptors have been identified in vertebrates. The TGF-β ligand first binds to TβRII, which promotes phosphorylation of TβRI, thereby transmitting intracellular signals in a Smad-dependent or -independent manner [63]. This intracellular TGF-β signaling operates by two alternative mechanisms based on the association of Smad proteins, as follows.

### 3.2. Smad-Dependent Signaling

There have been eight Smad proteins identified in vertebrates, called Smad 1-8. Smads 1, 2, 3, 5, and 8 are referred to as the receptor-associated Smads or R-Smads. In response to BMP or GDF activation, R-Smad1/5/8 is phosphorylated by ALK1//2/3/6, while R-Smad2/3 is activated by TGF-β, NODAL, or activin signaling and phosphorylated by ALK4/5/7 [64]. After activation, TGF-β binds to TβRII, which recruits and activates ALK5, which phosphorylates R-Smad2/3. After phosphorylation, R-Smad2/3 forms a heterodimeric complex with Smad4 (known as the common mediator Smad or co-Smad) and subsequently translocate to the nucleus (Figure 1) [65]. In the nucleus, the Smad complex acts as a transcription factor that can bind to chromatin to cause structural modifications. The Smad complex also associates with other transcription factors to achieve a strong binding affinity for Smad-binding elements in the promoters of the TGF-β target gene [66]. These Smad binding partners include various families of transcription factors, such as AP1, forkhead, homeobox, zinc finger, and basic helix-loop-helix. In addition, the Smad complex recruits co-activators, such as p300 and CREB-binding protein, or co-repressors, such as retinoblastoma-like 1 protein, to regulate gene transcription [65,67]. Although Smad proteins can serve as transcription factors, the functional outcome of the target gene relies on the transcriptional partners associated with Smads [68]. In a recently identified mechanism of Smad-based signaling, ALK5 activates R-Smad1/5 to cause TGF-β–induced anchorage-independent growth and cell mobility. Moreover, TβRI ALK1, expressed mainly by endothelial cells, can alternatively activate R-Smad1/5/8 [59,69].

### 3.3. Smad-Independent Signaling

TGF-β also interacts with alternative signal transducer proteins in a Smad-independent manner [61,63,70,71]. The TGF-β receptor-activated complex can activate non-Smad signaling pathways such as MAPK, PP2A/p70^s6k^, RhoA, and TAK1/MEKK1. Studies in Smad4-deficient cells have shown that TGF-β induces the MAP kinase pathway in a Smad-independent manner. Activation of the MAP kinase pathway in turn activates extracellular signal-regulated kinases (Erk1 and 2), p38, and JNK MAP kinases [63,70,71]. Activation of the Erk MAP kinase complex activates Ras to its GTP-bound form, and the kinase cascade includes c-Raf, Erk1 or Erk2, and MEK1 or MEK2. This protein complex activated by TGF-β also induces activation of the p38 and JNK MAP kinase pathway through the TRAF6 and TAK1. TRAF6 interacts with the TGF-β receptor complex, auto-ubiquitylates, and becomes active. Active TRAF6 binds with TAK1, causing polyubiquitylation and phosphorylation of TAK1. Activated TAK further activates the p38 MAP kinase and JNK [63,70,71,72]. Moreover, TGF-β receptor complexes interact with the Par6 (a polarity protein) and occludin (a tight junction protein) at epithelial cell junctions. TGF-β receptor-mediated phosphorylation of Par6 causes its association with Smurf1 to form the Par6-Smaruf1 complex, which confers ubiquitylation of RhoA and resultant dissociation of tight junctions. Interaction of occludin with TβRI is essential for the localization of TβRII to tight junctions. This localization is important in TGF-β–mediated EMT [63,70,71]. Thus, a combination of Smad mediated canonical and Smad-independent non-canonical TGF-β signaling cascades is necessary for cellular responses to internal and external stimuli.

## 4. TGF-β: A Regulator of Epithelial-Mesenchymal Transition

EMT is a crucial event in the progression of cancer and metastasis. TGF-β–mediated Smad-dependent and Smad-independent signaling induces transcriptional factors Snail, Slug, Twist, ZEB1, and ZEB2, which are critical in the regulation of EMT [73]. Repression of promoters of E-cadherin and occludin (factors responsible for epithelial phenotype) is the result of the actions of TGF-β, where the Smad3/4 complex can directly bind to the regulatory region of the Snail promoter to induce its transcription [73,74]. TGF-β–Smad signaling is a regulator of the double-negative loop that involves ZEB and miR-200 family transcription factors that balance the epithelial and mesenchymal states. TGF-β–Smad signaling has been shown to directly correlate with ZEB, but inversely correlate with miR-200 [75]. TGF-β signaling regulates proteases such as MMP2 and 9 and extracellular matrix components such as fibronectin and collagens [76]. Moreover, TGF-β through the Smad/RhoA pathway directly regulates EMT via a group of microRNAs that can change cytoskeletal recognition and epithelial polarity [77]. TGF-β signaling pathways such as the PI3K/Akt/mTOR pathway increase protein synthesis and cell motility and invasion during EMT in a Smad-independent manner. TGF-β also induces EMT through ubiquitylation and sumoylation, by which the Smad3/4 complex regulates the expression of HDM2, increasing the ubiquitylation and degradation of p53, thus inducing EMT progression [78]. Finally, TGF-β signaling represses the expression of the SUMO E3 ligase PIAS1, reducing the levels of sumoylated SnoN, an antagonist of TGF-β–mediated EMT [73,79].

## 5. Pleiotropic Role of TGF-β in Cancer Progression

Through its ability to promote cell cycle arrest, differentiation, and apoptosis, TGF-β prevents the abnormal proliferation of endothelial, epithelial, and hematopoietic cells, thus maintaining overall tissue homeostasis [80,81]. However, several cancer cells circumvent the suppressive effects of TGF-β by inactivation of essential components of the TGF-β signaling pathway, such as receptors or Smad, or downstream signaling alterations, through the interference of other signaling pathways. As a result, tumors lose their capacity to respond to TGF-β–mediated growth inhibition and apoptosis, leading to tumor angiogenesis, migration, and invasion and triggering tumor progression [59,82]. TGF-β can also modulate the tissue microenvironment in favor of cancer cell survival and propagation (Figure 1) [59]. Overall, dysregulation of TGF-β signaling reverses its normal role as a tumor suppressor and transforms it into a tumor inducer both by direct modulation of tumor cells and by indirectly modulating the host microenvironment [59,67].

In various biological processes, TGF-β induces opposing responses to cancer. For example, at the early stages of cancer, TGF-β suppresses tumor growth. In contrast, in the late stage of the disease or in metastatic tumors, TGF-β enhances tumor growth and progression of metastasis [67,83,84]. In metastatic tumors, TGF-β induces EMT, which leads to increased invasion and metastatic colonization at distant sites. The dual effects of TGF-β are widely known, but it is unclear when and how TGF-β switches from tumor suppressor to tumor inducer. Emerging research show that contextual functionality determines the dual role of TGF-β [85]. Recent evidence has identified miR-106b as a determinant of the molecular switch that determines TGF-β’s effects in the cell. miR-106b is elevated in late-stage tumors, where TGF-β increases transcription of miR-106b. This upregulation of miR-106b counterbalances the tumor-suppressing effects of TGF-β and enhances cell proliferation [86]. Another study showed that reprogramming of triple-negative breast cancer cells with overexpression of *GATA3* reduced TGF-β response, reverted EMT, converted cells from basal to luminal phenotype, and restored TGF-β–mediated anti-proliferative function [87]. Moreover, a mutation in p53 also switches TGF-β from tumor suppressor to inducer of metastasis [88]. In prostate cancer cells, PPARδ, a TGF-β target gene, plays a critical role in regulating the dual function of TGF-β. Repression of PPARδ increases tumor cell response to TGF-β–mediated growth inhibition, and activation of PPARδ promotes TGF-β–mediated tumor growth, invasion, and migration [89]. In addition to changes in molecular response, changes in the cellular microenvironment could also contribute to the bidirectional role of TGF-β in early versus advanced tumors. Changes in tissue stiffness and matrix rigidity could also influence functional response to TGF-β. Matrix rigidity does not directly change Smad signaling, but it modulates the PI3K/Akt pathway, which plays a vital role in EMT response and apoptosis. Likewise, high rigidity contributes to EMT, and low rigidity increases TGF-β–induced apoptosis [90]. Altogether, the studies referenced above have shown that the dual role of TGF-β is mainly dependent on the interaction of downstream pathway molecules with other molecular pathways as well as based on the microenvironment [59,67,85]. Most of these studies have been in cell culture or animal models and can provide insight into the mechanisms of dual-function TGF-β. However, the tumor microenvironment in humans is even more complex, and the dual role of TGF-β adds to that complexity, which is a challenge for designing therapeutics for metastatic tumors. Further translational research focusing on therapeutics to treat TGF-β–mediated early and advanced tumors would be beneficial in clinical settings.

## 6. TGF-β Expression in Human Tumors

With tumor advancement, TGF-β loses its suppressive effects and transforms into an oncogene to promote tumor growth, resulting in TGF-β overproduction, as commonly observed in many solid tumors. Moreover, increased TGF-β production indicates that TGF-β can induce its own expression and, hence, creates a malignant autocrine loop that could increase cell proliferation [91]. TGF-β expression is mostly elevated in mammary tumors compared to normal breast tissue, and it tends to increase with tumor progression [92,93,94]. In breast cancer patients, TGF-β expression levels are associated with cancer prognosis and angiogenesis [95]. Plasma TGF-β_1_ concentrations correlate with disease stage and advancement. Plasma TGF-β_1_ is elevated in breast cancer patients at the time of diagnosis and is either reduced or still elevated after surgical resection, and continuously elevate after surgical resection depending on postoperative tumor status [96]. Plasma TGF-β_1_ concentrations have prognostic significance even after surgical resection: patients with normalized plasma TGF-β_1_ after surgery had a better disease prognosis than those with continued elevated levels [97]. Similarly, in prostate cancer patients, during tumorigenesis, prostate cancer cells become nonresponsive to the antiproliferative effects of TGF-β [98]. About 30% of prostate cancer specimens do not express TGF-β receptors. Reduced TGF-β receptor expression in prostate tumors is correlated with higher tumor grade, increased tumor stage, and poor prognosis [99]. The expression of TGF-β in the tumor also provides an insight of its metastatic potential of the tumor. Increased circulating or urine TGF-β concentrations are also associated with a worse prognosis in prostate cancer patients [100].

Increased plasma TGF-β and CXCL1 are similarly associated with poor prognosis, an increased number of circulating tumor cells, and the propensity of these cells to cause lung metastases in patients with breast cancer [101]. Plasma TGF-β is also a useful marker for the prediction of cancer progression and treatment response in metastatic disease. In addition, a highly significant association between TβRII expression and reduced overall survival has been reported in patients with estrogen receptor–negative breast cancer [102]. In 38 cancer patients’ immunohistochemistry data, TGF-β concentrations were increased in both primary and metastatic tumors, and higher TGF-β expression in tumor stroma was associated with increased mortality [103]. Tumor tissue extracted from 89 prostate cancer patients showed that 67% of tumors showed under expression of TGF-β mRNA. A similar lack of expression was noticed in the benign tumors. However, 92.3% of patients with overexpressed TGF-β had a poor prognosis [104].

Since multiple signaling pathways are involved in determining the pleiotropic nature of TGF-β in cancer progression and metastases, considering TGF-β alone as a predictor may not be sufficient. The involvement of TGF-β downstream signaling molecules may provide significant insight into disease prognostication. For instance, increased Smad4 expression is associated with better prognostic outcomes in breast cancer patients. In contrast, increased phosphorylation of Smad2 is associated with poor prognosis [105]. Similarly, loss of TβRII indicates poor prognosis in prostate cancer [100]. These studies indicate the importance of evaluating tumor expression of TGF-β and circulating TGF-β in the context of downstream signaling molecules for prognostic and treatment purposes. Regarding the outcome of metastasis, in addition to molecular interaction in the metastatic tumor, the host microenvironment is a major contributing factor for establishing a tumor at a distant site. Both breast cancer and prostate cancer cells have a high affinity to the bone, which is a rich source of TGF-β and provides fertile soil for tumor cells to proliferate [12].

## 7. TGF-β and Bone Microenvironment

Coordination of growth factors, cytokines, and cell adhesion molecules is essential in maintaining normal bone homeostasis. TGF-β is one of the most abundant growth factors in the bone matrix and uniquely coordinates the activity of bone cells to maintain normal bone homeostasis [14]. It is a critical factor that regulates the differentiation and function of both osteoclasts and osteoblasts [106]. Osteoclasts resorbs bone, and once the resorption process is complete, a reversal period follows, after which osteoblasts deposit a new bone matrix to fill the resorption cavity, a process known as coupling. The newly deposited collagenous matrix is mineralized following a resting phase. In coupling the activity of bone-forming and bone-resorbing cells, TGF-β integrates physical and biological stimuli to maintain bone homeostasis [106].

Bone-forming osteoblasts secrete TGF-β, which remains embedded in a latent form in a mineralized bone matrix [107,108]. In response to bone resorption by osteoclasts, TGF-β is activated and released, and then acts as a chemoattractant for osteoprogenitor cells, recruiting those cells to the site of new bone formation [109]. TGF-β regulates the formation of extracellular matrix proteins and proteases in bone, including alkaline phosphatase, collagen I, osteocalcin, osteopontin, and MMP-13 [110]. Initially, TGF-β stimulates bone matrix synthesis, and, in later stages, it inhibits terminal osteoblast differentiation and bone matrix synthesis by repression of Runx2 through a Smad3-dependent mechanism [107]. TGF-β also inhibits osteocyte apoptosis, partially via Smad3-dependent and vitamin D receptor-dependent mechanisms. Therefore, TGF-β plays a distinct role at each stage of the osteoblast life cycle [106,111].

In addition to its function in osteoblast recruitment and function, TGF-β plays a role in the differentiation of osteoclasts at different stages. TGF-β promotes the recruitment of osteoclast precursors to the bone site and stimulates their proliferation and differentiation [108,112]. Moreover, TGF-β can directly influence osteoclast function, as osteoclasts also express TGF-β receptors I and II [106]. During active bone resorption, osteoclasts secrete cathepsins, which proteolytically activate and release TGF-β from the latent complex. This activated TGF-β feeds back on osteoblasts. TGF-β acts on osteoblasts to regulate the recruitment of osteoclast regulatory proteins such as RANKL, OPG, ephrin B2, and ephrin B4 [113]. TGF-β also regulates bone mass and mechanical strength via its effects on osteocytes. Mechanical load rapidly represses the net activity of the TGF-β pathway in osteocytes, which reduces phosphorylation and activity of Smad2 and Smad3, thus resulting in the loss of anabolic response of bone to mechanical load, indicating that the mechanosensitive regulation of TGF-β signaling is necessary for load-induced bone formation [106,114]. It has also been shown that osteocyte-intrinsic TGF-β signaling regulates bone quality via osteocyte perilacunar/canalicular remodeling [115].

## 8. TGF-β and Tumor-Bone Interaction in Bone Metastases

Bone is a preferred site of dissemination for breast and prostate cancer cells. The unique bone microenvironment contains various growth factors, including TGF-β and growth-promoting agents, that attract tumor cells into bone and promote growth and progression of cancer in the bone microenvironment (Figure 1). Bone metastases are a frequent complication of advanced cancer, occurring in about 70–80% of breast and prostate cancer patients [8]. Most patients with bone metastases often suffer from debilitating skeletal-related events such as pain, pathological fractures, hypercalcemia, and nerve compression, which reduce the patient’s quality of life and survival [7,12,116]. The patterns of bone metastases in patients with breast and prostate cancer are different: breast cancer is mostly osteolytic in nature, and prostate cancer is either osteoblastic or a mixture of osteolytic and osteoblastic patterns [117].

Bone metastasis is a multistep process in which cancer cells must detach from their tissue of origin or primary site. After detachment, the cancer cell acquires a survival mechanism in the bloodstream. Disseminated cells in the circulation are attracted by the bone marrow and successfully invade it. They adopt a new microenvironment and secure themselves in the marrow by cell–cell or cell–matrix adhesion. After homing to the bone marrow and forming a colony, tumor cells release factors that cause osteolysis and release factors stored in the mineralized bone matrix [117,118]. These bone-derived factors feed tumor cells and expand the colony, which further secretes tumor-derived growth factors to cause further osteolysis, supporting tumor growth and progression in the bone microenvironment. The bi-directional interaction between bone cells and tumor cells is called the vicious cycle of bone metastases (Figure 1) [119]. Bone biopsies from patients with breast cancer bone metastases showed phosphorylated Smad2positive nuclear staining by histology in 75 of the patients, indicating active TGF-β signaling [120]. Owing to its critical role in bone remodeling, TGF-β is a crucial component of this cycle that drives bone destruction and progression of tumor growth [119]. Release of TGF-β from the bone matrix by osteoclastic bone resorption induces tumor production of osteolytic factors such as interleukin 11, connective tissue growth factor, MMP-1, CXCR4, and parathyroid hormone–related peptide [121].

TGF-β also plays a vital role in the progression of prostate cancer bone metastases. In a mouse model of prostate cancer bone metastases, blockade of TGF-β signaling in prostate cancer cells inhibited osteoblastic bone metastases and reduced tumor burden [122]. Gene expression analysis of prostate cancer cells showed that TGF-β stimulated the expression of several genes involved in bone metastasis. Prostate transmembrane protein androgen induced 1 (*PMEPA1*) was a highly upregulated gene that TGF-β transcriptionally induced. Although *PMEPA1* was induced by TGF-β, membrane-bound PMEPA1 inhibited TGF-β signaling and prostate cancer bone metastases in a negative feedback loop. This study has also indicated that low expression of PMEPA1 is associated with poor prognosis in breast and prostate cancer and can serve as a prognostic marker for bone metastases [123].

## 9. TGF-β and the Immune System in the Tumor Microenvironment

**Figure 2:**
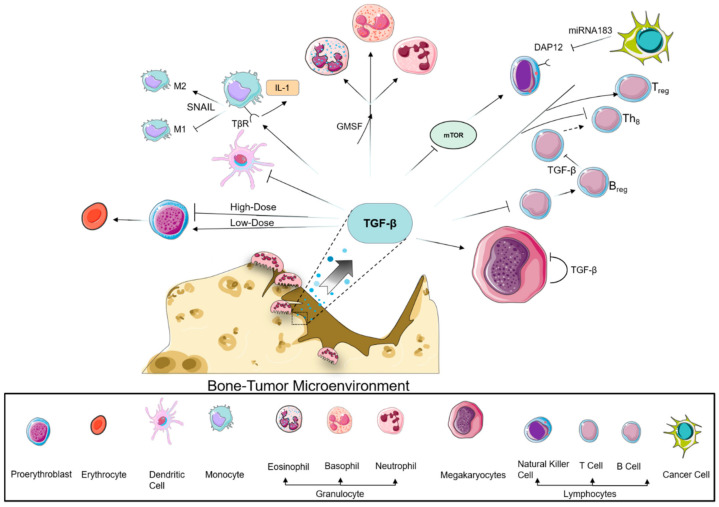
Role of TGF-β in immune cells of the marrow. Excess release of TGF-β from the bone matrix exerts a multitude of interactive effects on the surrounding immune cell populations within the bone marrow. Erythropoiesis is affected in a polarized fashion, where TGF-β induces red blood cell proliferation at low concentrations, while higher concentrations are inhibitory to proerythroblasts. TGF-β upregulates granulocyte differentiation and binds to its surface receptor on monocytes to drive M2 maturation via Snail while suppressing the M1 macrophage phenotype. Lymphoid maturation is blockaded through multiple pathways. Bone-derived TGF-β and autocrine synthesized TGF-β suppress the function and proliferation of megakaryocytes. Differentiation of immature lymphoblasts is suppressed, preventing proliferation of B and T cells but driving the T_reg_ phenotype. Additionally, TGF-β inhibits mTOR, which is necessary in natural killer cell recognition of cancer cells.

Each TGF-β isoform exhibits a profound and varied effect on the regulation of hematopoiesis via autocrine or paracrine mechanisms which may initiates the interactions between tumor cells and immune system [124]. The immune system can inhibit the growth of some cancers through a process known as immunosurveillance. During metastasis to distant organs, cancer cell survival relies on the cancer’s ability to escape detection by the immune system using multiple mechanisms. Many metastatic cancer cells can achieve immune suppression by targeting immune checkpoint pathways; TGF-β can contribute to this immune suppression by enhancing cancer cell invasion. A balance between immunity and antigen tolerance is required for the proper function of the immune system, which depends on the dynamic regulation of numerous cell fate transitions to produce various lymphocyte subtypes in adequate numbers. TGF-β is a pivotal and pleiotropic regulator of immune responses, assuming many critical roles regulating immune response while altering immunity under differing conditions [125]. For instance, TGF-β acts as a potent immunosuppressive cytokine through effects on both cell differentiation and cell proliferation. Immune cells that control cancer are divided into two main categories, the myeloid and lymphoid lineages. TGF-β regulates the generation and effector functions of many immune cells across these two hematopoietic subtypes (Figure 2) [126,127].

### 9.1. Myeloid Compartment

Monocytes, macrophages, and dendritic cells: Early work has demonstrated that peripheral cells of the monocyte lineage are drawn to low concentrations (0.1–10 pg./mL) of TGF-β. When TGF-β is present at high concentrations (1 ng/mL), TGF-β surface receptors on monocytes induce growth factor (i.e., IL-1) production [128], which can increase fibroblast proliferation [129]. Inactivated monocytes secrete TGF-β, albeit in low concentrations; however, TGF-β has been demonstrated to play a role in activating the M2 macrophage phenotype [130] via Snail-mediated suppression of the M1 (pro-inflammatory) phenotype [131] (Figure 2). Conversely, TGF-β inhibits the generation of antigen-presenting dendritic cells and their ability to activate T cells through inhibition of co-stimulatory surface molecules [132], despite their abundance [133,134]. In contrast, TGF-β_1_ is distinctly necessary to induce activation of epithelial-bound Langerhans cells, a type of dendritic cell.

Erythrocytes: TGF-β also has an inhibitory effect on erythropoiesis through blockade of erythroid progenitor proliferation [135]. This relationship is exemplified by TGF-β inhibitors’ stimulation of red blood cells by enhancing burst forming unit-erythroid progenitors [136]. However, conflicting data have indicated a dose-dependent relationship where low-dose TGF-β_1_ enhances rapid red blood cell formation from hematopoietic stem cell differentiation, in contrast to the inhibitory effects elicited by high-dose TGF-β (Figure 2) [137].

Megakaryocytes: Cancer cells can avoid contact with immune cells by forming aggregates with platelets in the blood. TGF-β can be produced by megakaryocytes but then inhibits further megakaryopoiesis (Figure 2) [138].

Granulocytes: TGF-β regulates the complex behavior of macrophages and neutrophils, thus forming a network of negative immune regulatory inputs. In combination with granulocyte-monocyte colony-stimulating factor, TGF-β_1_ increases granulopoiesis in vivo and in vitro [139]. Blocking TGF-β can dramatically shift the phenotype of neutrophils from pro-tumor to a CD11b^+^/Ly6G^+^ tumor-associated neutrophil that exhibits cancer cell toxicity [140].

### 9.2. Lymphoid Compartment

T cells: Cancer cells often express programmed cell death ligand (PD-L1), which binds to the PD-1 receptors on T cells to inhibit their activation [141]. TGF-β controls adaptive immunity by directly promoting the expansion of T_reg_ cells and inhibiting the generation and function of effector T cells (Figure 2). Blockade of TGF-β negatively affects CD8 T-cell activation, driving immune evasion by blocking the generation of the Th1 T-cell phenotype [142] and restricting T cells from infiltrating the tumor [143].

B cells: Humoral responses are associated with immune protection in multiple human cancers. For instance, dense B-cell infiltration has been correlated with better clinical outcomes in human hepatocarcinoma [144], breast cancer [145], and cutaneous melanoma [146], which orchestrates mechanisms to decrease B-cell infiltration. TGF-β is a potent regulator of B-cell proliferation, survival, activation, and differentiation. TGF-β inhibits the proliferation of mature human and immature murine B cells. Exogenous administration of TGF-β regulates B-cell activation by inhibiting immunoglobulin synthesis and class switching of most IgG isotypes [147,148]. Regulatory B cells, which play a role in promoting T_reg_ cells, also secrete TGF-β (Figure 2) [149]. Together, these observations summarize the complex and intricate role TGF-β plays in adaptive immunity.

Natural killer cells: The physical nature of these cells protects cancer cells against perforin/granzyme-mediated natural killer cell cytotoxicity [150]. TGF-β similarly controls the innate immune system by inhibiting natural killer cells through repression of mTOR [151]. The significance of this interaction is demonstrated when natural killer cells are devoid of TGF-β, a condition that permits increased anti-tumor activity. TGF-β also inhibits CD16-induced production of IFN-γ by natural killer cells through phosphorylation of Smad3 [152]. Additionally, miR-183 induced by TGF-β represses the natural killer cell surface receptor-dependent DAP12, thereby reducing tumor cytolysis [153].

## 10. Therapeutic Approaches to Target TGF-β Signaling

With the known contribution of TGF-β signaling in cancer, the inhibition of TGF-β is expected to attenuate cancer progression. However, the dual nature of TGF-β signaling in cancer poses a significant challenge for the use of anti–TGF-β therapies. A big question is which arm of TGF-β function to target: the tumor-promoting arm or the tumor inhibitor arm? Careful selection of the patient population in whom to use such agents to treat metastatic disease may require preventing harmful effects of anti–TGF-β therapeutics [19,20,154,155]. Despite this challenge, TGF-β signaling inhibition provides multiple therapeutic opportunities. The major classes of TGF-β inhibitors that have been investigated include: (1) monoclonal antibodies that prevent TGF-β binding to receptor complexes; (2) small-molecule inhibitors of TGF-β receptor kinases, which inhibit TβRI/ALK5 (and TβRII) kinase activity and prevent downstream signaling; (3) antisense oligonucleotides, which can suppress TGF-β expression at the transcriptional/translational level; (4) ligand traps, which are soluble, high-affinity ligand traps that can prevent TGF-β from binding to its receptors. These inhibitors comprise Fc-stabilized dimers of TβRII extracellular domains that are designed to sequester TGF-β_1_ and TGF-β_3_ but not TGF-β_2_ and thus prevent their signaling [19,154,155,156].

### 10.1. Anti–TGF-β Antibodies and TGF-β Receptor Inhibitors

TGF-β expression in the tumor and its circulating levels and activity of TGF-β downstream signaling molecules often increase during cancer progression, and these levels are correlated with aggressive tumor phenotypes such as increased grade and advanced stage [157,158]. Normalization of the amount of active TGF-β may reduce disease progression, by either reducing the concentration of TGF-β itself or blocking its action. Reduction in active TGF-β signaling can be carried out by different mechanisms, such as a TGF-β ligand trap, which uses a soluble decoy receptor made up of the TβRII or TβRIII ectodomain, or via TGF-β–neutralizing antibodies [19,20,154,155,156].

Several antibodies against TGF-β are currently in development or clinical trials for safety and potential clinical use in cancer patients. Pan-neutralizing mouse monoclonal antibodies have been developed to target individual ligands or all three TGF-β isomers. Anti–TGF-β monoclonal antibodies 1D11, 2G7, lerdelimumab, metelimumab, and fresolimumab (GC1008) neutralize all three TGF-β isoforms and demonstrate significant antimetastatic activities in mouse models of breast cancer [19]. 1D11 has also been shown to reduce MDA-MB-231 tumor burden and osteolysis and to increase bone volume [19,154,155,156,159]. Similarly, 2G7 also showed antitumor activity in mouse models [160]. Fresolimumab has been tested in a phase I trial involving 28 patients with malignant melanoma and one patient with renal cell carcinoma [19]. Of these patients, only few showed partial response; major adverse effects were seen in four patients who developed reversible cutaneous keratoacanthomas or squamous cell carcinomas and one patient who acquired hyperkeratosis [19,161]. Additional clinical trials with fresolimumab were initiated in glioma and in combination with radiotherapy in metastatic breast cancer [19,154,155,156,160].

The trivalent TGF-β receptor trap comprises one extracellular domain of betaglycan flanked by two extracellular domains of TβRII. This ligand trap has been shown to efficiently block the interaction between TGF-β and its receptor [20,154,155,156,162]. TGF-β binding to its receptors can also be prevented using recombinant Fc-fusion proteins containing the soluble ectodomains of TβRII or TβRIII. It has been shown that these biologically active compounds can reduce breast cancer and lung metastases in animal models without any significant side effects [154]. Preclinical studies showed that expression of TβRII or TβRIII via recombinant DNA prevents their binding to TGF-β and thus inhibits metastasis in mouse models of liver, breast, and pancreatic cancers [20,154,155,163,164].

### 10.2. TGF-β Receptor Kinase Inhibitors

TGF-β receptor I (ALK5) transmit a signal to the type II receptor, which initiates the Smad cascade to transmit the TGF-β signaling response. This pathway has been extensively investigated with compounds that can inhibit tumor progression. TGF-β receptor kinase inhibitors are small-molecule inhibitors that inhibit kinase catalytic activity of TβRI/ALK5 via the ATP-binding domain of TGF-β receptor kinases [19,154,155,156]. There are advantages to the development and scalability of small-molecule inhibitors, but the lack of selectivity of kinase inhibitors is potentially problematic. Currently, all known small-molecule TβR1/ALK5 inhibitors have shown similar efficacy against ALK4 kinase activity but limited efficacy against ALK7 [19,154,156,165,166,167].

Kinase inhibitors which targets the ATP-binding sites of TβRI/ALK5 have been widely studied and have included SD-208 and SD-093 (Scios, Titusville, Hopewell Township, NJ, USA), SB-431542 (GlaxoSmithKline, Brentford, UK) [165], Ki26894 (Kirin Brewery Company, Tokyo, Japan), and LY364947 (Eli Lilly and Company, Indianapolis, IN, USA) [168]. Each of these compounds blocks receptor kinase activity and inhibits proliferation, invasion, or metastasis of tumor cells in animal models of breast cancer, glioma, and pancreatic cancer [19,154,155,166,167]. In an intracardiac mouse model of human breast cancer cells, MDA-MB-231, when used in a preventive protocol, SD-208 significantly decreased osteolytic lesion areas, inhibited bone metastatic tumor growth, and increased survival [154]. Furthermore, in mice who already had established bone metastases, SD-208 inhibited further tumor progression and, hence, reduced the formation of osteolytic lesions [169]. Non-tumor mouse models treated with SD-208 had increased bone mass, strength, and material quality [114], which could benefit patients who have disease-related bone loss [114]. SD-208 treatment has also been shown to reduce cancer-associated bone pain [170]. LY364947 is a dual inhibitor of TβRI and TβRII shown to inhibit metastatic activity in several mouse models of colon, pancreatic, breast, and prostate cancer [19,20,154,156].

### 10.3. Inhibitors of TGF-β Synthesis

Antisense oligonucleotides (ASOs) block synthesis of active TGF-β and thus decrease its availability to the local tumor microenvironment. ASOs are single-stranded polynucleotides 13–25 nucleotides long that hybridize to complementary RNA to inhibit mRNA function and subsequent protein biosynthesis through modulation of splicing and mRNA degradation by RNase H [20,154,155,156,171,172]. The role of autocrine TGF-β in metastasis formation was interrogated using a PyMT orthotopic model of mammary tumors [173,174]. Overexpression of TGF-β by the PyMT tumors resulted in increased metastasis and reduced survival; however, overexpression of a TGF-β ASO decreased metastasis and improved survival [173,174]. An 18-mer ASO called AP12009 (trabedersen) that targets TGF-β_2_ expression in glioma, pancreatic carcinoma, and malignant melanoma has been tested in a phase III clinical trial for astrocytoma showed better survival outcomes in astrocytoma patients [19,154,155,156,175].

### 10.4. Additional TGF-β Antagonists

Other biologically active molecules that neutralize TGF-β or its downstream signaling have also been reported. Our publication shows that the natural plant-based compound halofuginone inhibits TGF-β signaling in vitro in several cell types and in in vivo models [176,177]. In intracardiac mouse models of 1205Lu melanoma cells, which metastasize to bone, and MDA-MB-231 breast cancer metastases, systemic daily treatment with halofuginone significantly inhibited the formation of osteolytic lesions and bone metastases [176,177]. However, a phase II clinical trial testing halofuginone as sarcoma treatment did not show clear clinical benefits [178].

Small protein molecules called “peptide aptamers” inhibit TGF-β signaling by specifically binding to specific proteins. Peptide aptamers consist of two domains, a target-binding domain and a scaffolding domain that stabilizes resultant molecules [19]. Peptide aptamers block Smad2 and Smad3, preventing the recruitment of Smad4 [154]. Cui et al. developed three classes of peptide aptamers based on their Smad-interacting motifs CBP, FoxH1, and Lef1, which were able to reduce TGF-β expression [179]. The Trx-SARA aptamer has been shown to inhibit Smad2/3 complex formation with Smad4, and subsequent inhibition of TGF-β induced EMT in a mouse epithelial cell line [19,154,180].

## 11. Enhancing TGF-β Inhibitors’ Effects by Combination with Other Therapies

A combination therapy approach of using TGF-β inhibitors with other therapies can be more effective in treating bone metastases compared to single-agent treatment. For example, bisphosphonates indirectly prevent TGF-β release from the bone by preventing bone resorption, and thus bisphosphonates combined with TGF-β inhibitors can have greater therapeutic effectiveness than either agent alone. Similarly, cytotoxic chemotherapies such as rapamycin and doxorubicin, if combined with TGF-β inhibitors, could improve efficacy [181,182]. Our laboratory showed that SD-208 combined with anti-resorptive zoledronic acid reduced the progression of established osteolytic metastases from breast cancer more effectively than either therapy alone [178]. Similarly, the combination of halofuginone and zoledronic acid significantly reduced bone metastases and tumor burden in a mouse model of MDA-MB-231 bone metastases [176]. Using the same bone metastasis model of intracardiac inoculation of MDA-MB-231 breast cancer cells, the combination treatment of SD-208 and an inhibitor of HIF-1α (methoxyestradiol-2) was associated with significant reduction in tumor burden and osteolysis compared to single-agent therapeutics [156,169].

The PD-1 receptor and the PD-L1 ligand can be utilized as therapeutic targets via monoclonal antibodies that block the interaction of PD-1 on T cells with PD-L1 on tumor cells [19,154]. Targeting this pathway has led to significant improvement in tumor response in patients. However, some patients showed only marginal improvement after antibody treatment. The expression of TGF-β can explain the differences in the treatment response as a possible mechanism. TGF-β plays a role in immune system homeostasis, and overexpression of TGF-β inhibits the function of cytotoxic T cells and natural killer cells, inhibits maturation and antigen presentation by dendritic cells, increases the production of T_reg_ cells, and thus leads to immune suppression [19,154]. Hence, a combination of a TGF-β inhibitor and a PD-1/PD-L1 inhibitor could potentially improve immune response and augment the effects of the PD-1/PD-L1 inhibitor [154,156]. A pan–TGF-β antibody (SAR439459) as a single agent and in combination with anti–PD-1 antibody (cemiplimab) is currently in a clinical trial for advanced solid tumors [19,156]. It was shown that co-administration of TGF-β–blocking and anti–PD-L1 antibodies reduced TGF-β signaling in stromal cells, facilitated T-cell penetration into the center of tumors, and provoked vigorous anti-tumor immunity and tumor regression [143].

In a clinical trial for metastatic breast cancer, patients who had at least three distinct sites of metastasis and tumor progression after the first line of therapy were randomized to receive 1 mg/kg or 10 mg/kg of the TGF-β–blocking antibody fresolimumab every 3 weeks for five cycles, with focal radiotherapy to a metastatic site at week 1 (three doses of 7.5 Gy). The TGF-β blockade during radiotherapy was feasible and well tolerated. Patients receiving the 10 mg/kg dose had a favorable systemic immune response and experienced longer median overall survival than the 1 mg/kg dose group [183]. A follow-up publication from the same study reported that T-cell functionality was hampered in a way that was at least partly mediated by the PD-1 signaling pathway in these patients with metastatic breast cancer. These preliminary data support the rationale for investigating the possible beneficial effects of adding PD-1 blockade to improve responses to TGF-β blockade and radiotherapy [184].

While immune checkpoint therapy shows encouraging results in patients with metastatic castration-resistant prostate cancer (CRPC), it shows a suboptimal response against bone metastases. In a mouse model of bone CRPC by intraosseous injection of syngeneic Myc-CaP prostate cancer cells, there was no effect of immune checkpoint therapy despite a significant effect on subcutaneous tumor growth. However, blocking TGF-β along with immune checkpoint therapy increased Th1 cells and subsequent regression of bone CRPC [25]. Nonetheless, it was suggested that PD-1 blockade might have a protective function against bone destruction caused by cancer. In a recent study, PD-1^−/−^ mice demonstrated remarkable protection against bone destruction induced by femoral inoculation of Lewis lung cancer cells [185].

Activation of CD8+ T cells is an evolving concept in cancer immunotherapies. Genetically engineered chimeric antigen receptor (CAR)-expressing CD8+ T cells have been used to enhance antitumor effects [186]. Since TGF-β inhibits cytotoxic T-cell activation, inhibition of TGF-β is necessary for an effective response using CAR-T–based therapies. In newer therapeutic modalities, modifications of CAR-T–based approaches led to the engineering of T cells that express CAR with an extracellular, variable fragment of TGF-β–neutralizing antibody that can cause TGF-β–driven dimerization of the TGF-β–binding CAR, resulting in activation of these T cells. Using this approach instead of immunosuppression, TGF-β can behave as a potent enhancer of these CAR-T cells [187,188].

## 12. Challenges of TGF-β Targeting as Therapeutics

Due to the broad expression of TGF-β in most tissues and its overall cell growth regulatory effects, blockade of TGF-β or its downstream signaling provides promising therapeutic opportunities for treating many different disease conditions. However, the mechanism of the oncogenic and tumor suppressor role (dual role) of TGF-β in cancer is poorly understood. Thus, the development of specific TGF-β antagonists is challenging. Additionally, the contribution of excess bone-derived TGF-β in to the progression of bone metastases poses additional challenge in the use of TGF-β signaling as a therapeutic target As TGF-β is secreted by nearly all cell types and regulates several critical biological processes in normal cells, inhibition of TGF-β can have potential adverse off-target tissue-specific effects [22]. Together with the pleiotropic actions of TGF-β and lack of biomarkers for patient selection criteria, dosing requirements and treatment decisions are still under the developmental stage. Targeting TGF-β signaling is not aimed at killing cancer cells but aimed at inhibiting invasion and metastases; hence, TGF-β antagonists have to be used in combination with other therapeutics that kill cancer cells [162,189]. Although anti–TGF-β therapies were successful in vitro and in preclinical models, clinical trials showed minimum effects and considerable side effects [190,191]. Potential off-target tissue toxicity and poor drug delivery to tumor cells in the setting of bone metastases could possibly be overcome with the use of bisphosphonates, which have anti-tumor effects and can also prevent TGF-β release [192]. A conjunction of small-molecule inhibitors with bisphosphonates could provide an alternative approach in targeting TGF-β. Since these strategies can allow long exposure of TGF-β inhibitors to the bone microenvironment, they could have greater therapeutic efficacy than a shorter exposure [193]. The combination of anti–TGF-β therapies with immune checkpoint inhibitors is another promising approach that could have a prolonged anti-tumor response. Simultaneously targeting TGF-β and PD-1/PD-L1 could help improve the poor outcomes of anti–TGF-β therapies in clinical trials [191,194,195,196,197]

## 13. Conclusions

TGF-β has been identified as a key regulator of bone metastases owing to its function as a pluripotent cytokine in tumor, bone, and the immune system. TGF-β transmits cellular signaling through Smad-dependent or Smad-independent signal transmitters. In bone metastases, TGF-β regulates a feed-forward vicious cycle of tumor growth in bone in favor of osteolysis. One of the critical events in metastasis is EMT, regulated by TGF-β–driven changes in molecular pathways responsible for EMT. Although TGF-β is a major regulator of tumorigenesis and TGF-β signaling is present in nearly all cells, an ambiguous function of TGF-β as a tumor suppressor in early-stage tumors and a tumor enhancer in advanced cancer poses a challenge in developing therapeutics that target TGF-β for the treatment of breast and prostate cancer metastases. TGF-β pathway–targeting therapeutics such as TGF-β antibodies, TGF-β receptor kinase inhibitors, and TGF-β antisense oligonucleotides have been tested in preclinical animal models and clinical trials in cancer patients, including patients with bone metastases. However, toxicities associated with these TGF-β–targeting agents currently prevent their use in patients. A therapy approach combining TGF-β inhibitors with bone-targeted agents may have more beneficial effects than single agents and overcome some drug limitations. In the current era of immunotherapy, combination therapeutic approaches of combining TGF-β inhibitors with PD-1/PD-L1–mediated inhibition or with cell-based therapy such as CAR-T are in progress. Owing to the abundance of TGF-β in bone, the neutralization of TGF-β with anti–TGF-β antibodies could make immune checkpoint inhibition more effective in solid tumors with bone metastases. The promising results of first clinical trials using a synergistic therapeutic approach of targeting TGF-β with immunotherapy should encourage continued testing of these modalities to overcome the limitations of TGF-β pathway inhibition.

## Data Availability

Not applicable.

## Abbreviations

TGF-β	Transforming growth factor β
OPG	Osteoprotegerin
PD-L1	Programmed cell death ligand -1
IGF	Insulin-like growth factor
TNFs	Tumor necrosis factors
ILs	Interleukins
RANKL	Receptor activator of NF-κB ligand
IL-1	Interleukin-1
MMP	Matrix metalloproteinases
GDF	Growth differentiation factor
ALKs	Activin-receptor like kinases
MAP	Mitogen-activated protein
JNK	c-Jun amino-terminal kinase
TRAF6	TNF receptor-associated factor 6
ZEB	Zinc finger E-box-binding homeobox
DAP12	DNAX activating protein 12 kDA
CBP	CREB-binding protein
FoxH1	Forkhead box protein H1
Lef1	Lymphoid enhancer factor 1
CAR	Chimeric antigen receptor
PMEPA1	Prostate transmembrane protein androgen induced 1
